# Rhythm and rate control strategies in patients with long-standing persistent atrial fibrillation treated with cardiac resynchronization: the results of the randomized Pilot-CRAfT study

**DOI:** 10.1007/s00392-024-02541-z

**Published:** 2024-10-10

**Authors:** Jan B. Ciszewski, Mateusz Tajstra, Ilona Kowalik, Aleksander Maciąg, Tomasz Chwyczko, Agnieszka Jankowska, Edyta Smolis-Bąk, Bohdan Firek, Dariusz Zając, Jarosław Karwowski, Hanna Szwed, Mariusz Pytkowski, Mariusz Gąsior, Maciej Sterliński

**Affiliations:** 1 2nd Department of Cardiac Arrhythmia, Centre of Cardiac Arrhythmia, National Institute of Cardiology (Narodowy Instytut Kardiologii Stefana kardynała Wyszyńskiego Państwowy Instytut Badawczy), 42 Alpejska Street, 04-628 Warsaw, Poland; 2 3rd Department of Cardiology, School of Medicine with the Division of Dentistry in Zabrze, Silesian Center for Heart Diseases, Medical University of Silesia, Zabrze, Katowice Poland; 3 National Institute of Cardiology, Warsaw, Poland; 4 Department of Coronary Artery Disease and Cardiac Rehabilitation, National Institute of Cardiology, Warsaw, Poland; 5 Present Address: Department of Cardiac Surgery, Medical University of Warsaw, Warsaw, Poland; 6 Present Address: Department of Heart Diseases, Postgraduate Medical School, Warsaw, Poland; 7 1st Department of Cardiac Arrhythmia, Centre of Cardiac Arrhythmia, National Institute of Cardiology, Warsaw, Poland

**Keywords:** Cardiac resynchronization, Biventricular paced beats percentage, Atrial fibrillation, Electrical cardioversion, Heart failure

## Abstract

**Background:**

Atrial fibrillation (AF) is common in cardiac resynchronization therapy (CRT) recipients. It is a marker of impaired CRT response mainly mediated by the reduction of effectively captured biventricular paced beats (BiVp). There are no randomized trials comparing strategies to maintain high BiVp percentage.

**Objective:**

To compare the efficacy of rhythm vs rate control strategies in CRT recipients with long-standing persistent AF.

**Methods:**

We performed a randomized trial including CRT recipients with persistent AF resulting in low BiVp%. All patients received amiodarone, the rhythm control group received external electrical cardioversion (EC), and the rate control group received atrioventricular node ablation, if needed. The primary end-point was 12-month BiVp% (NCT).

**Results:**

43 patients were included in the analysis. The mean age was 68.4 (SD: ± 8.3) years and the mean BiVp% 82.4% ± 9.7%. AF lasted 25 ± 19 months. The mean baseline left ventricular ejection fraction (LVEF), left atrium area, and the maximal oxygen uptake (VO2max) were: 30 ± 8%, 33 ± 7 cm^2^, and 14 ± 5 mL/(kg*min), respectively. The EC success rate was 58%. 38% patients remained in sinus rhythm (SR) after 12 months. BiVp% increased similarly in both arms reaching 99% [95% CI 97.3–99.8] and 98% [94.0–99.0], *P* = 0.14 in rhythm and rate control groups, respectively. LVEF raised significantly only in the rhythm control group (ΔLVEF 4.1 (± 7.3), *P* = 0,018) which was driven by the patients who maintained SR. No differences in VO2max, QoL, clinical and safety end-points were observed.

**Conclusion:**

Despite comparable BiVp% in both groups, only restoration of SR led to improved left ventricular ejection fraction in CRT patients with long-standing AF.

**Trial registration:**

NCT01850277 registered on 22/04/2013.

## Background

Cardiac resynchronization therapy (CRT) is highly effective treatment in selected population of heart failure (HF) patients. It has been proven to reduce HF symptoms, improve exercise tolerance, lead to reverse remodeling, improve ejection fraction, increase the quality of life, lower the risk of HF hospitalization and reduce mortality [1]. However, the evidence supporting use of CRT in patients with atrial fibrillation (AF) is weaker and AF is regarded as a marker of poorer response to CRT [2–4]. The main factor to affect good response to resynchronization during AF is an inability to maintain adequate percentage of effectively captured biventricular paced beats (BiVp%) which should as close to 100% as possible, exceeding 95–98% [5, 6]

High BiVp% in AF patients may be obtained by two different treatment strategies. The first one is the rate control strategy whose aim is to slow down the ventricular rate during AF so as to increase BiVp%. The rate control obtained by pharmacotherapy is not always sufficient [7]. In such cases, atrioventricular node ablation (AVNA) should be considered. It is the only treatment option in CRT recipients with AF that is proven to maintain CRT efficacy similar to patients with sinus rhythm (SR) [8]. However, it leaves patients pacemaker dependent. The second one is the “rhythm control” strategy, which is of limited efficacy, while the HF presence is a well-established risk of AF recurrence. Nevertheless, the rhythm control strategy may initiate CRT-mediated reverse remodeling by ensuring adequate BiVp% and may help to get out of the HF-AF vicious circle. This hypothesis is supported by the fact that in CRT recipients undergoing atrioventricular node ablation (AVNA), a spontaneous rhythm resumption (SRR) is observed in up to 10% of the cases [9].

While AF is present in 25–50% of CRT recipients [10, 11] and permanent or persistent forms constitute half of the cases [12], the proper choice of AF treatment is of great importance. Surprisingly, there is lack of randomized studies comparing these treatment strategies dedicated to CRT patients. More research on CRT efficacy in AF patients (especially persistent AF) is needed which is clearly stated in the latest ESC guidelines on both heart failure [13] and cardiac pacing and cardiac resynchronization [6].

### Aim of the study

The purpose of this **C**ardiac **R**esynchronization in **A**trial **F**ibrillation **T**rial pilot study (Pilot-CRAfT) was to compare the efficacy of rhythm vs rate control strategies in the CRT recipients with long-standing persistent (or classified as “permanent”) AF resulting in inadequate BiVp%.

## Methods

### Inclusion criteria

Briefly, patients were eligible to enter the study if they were at least 6 month in atrial fibrillation, at least 3 months after CRT implantation, had atrial pacing lead implanted, had no contraindications to amiodarone, and had cumulative BiVp% lower or equal to 95%. Detailed inclusion and exclusion criteria were described previously [14]. However, during the enrollment period, the study definition of long-standing persistent AF was shortened from 1 year to 6 months due to the slow enrollment rate.

### Interventions

In both treatment arms, amiodarone was given orally, beginning with the loading dose of at least 8.4 g during the first 3–4 weeks. The rhythm control strategy consisted mainly of external electrical cardioversions (EC). Performing AF ablation procedure was possible but not obligatory. The rate control strategy included pharmacotherapy as the first-line treatment. If adequate BiVp% had not been obtained by the combination of AV node slowing agents, performing AVNA procedure was recommended, but the final qualification to AVNA was left to the therapeutic team’s discretion.

### Follow-up

The follow-up period lasted 12 months and consisted of 4 visits with the device control taking place every 3 months. On the baseline, the 3-month and the 12-month visits, the study participants underwent echocardiographic examination, cardiopulmonary exercise test (CPX) and were asked to fill the Minnesota Living with Heart Failure Questionnaire (MLHFQ)—a quality of life questionnaire dedicated to HF patients.

### Study end-points

The primary end-point was the cumulative BiVp% assessed on the 12-month visit. Among the secondary end-points were: the left ventricular ejection fraction (LVEF) assessment, the maximal oxygen uptake (VO2max) during CPX, the quality of life (QoL). We also analyzed device detected arrhythmic events and clinical end-points such as mortality, the number of planned and unplanned hospitalizations.

The study protocol was designed and performed in accordance with Declaration of Helsinki. The detailed study protocol has been published elsewhere [14] and the study was registered on the ClinicalTrials.gov webpage (NCT01850277). Study participants were enrolled and then followed-up in two high-volume centers in Poland. Local ethics committees relevant to both of the study centers approved the study protocol and were reported timely about the adverse events.

### Statistical analysis

Randomization was provided by an independent statistician with the use of independent permuted blocks for each study center.

The comparison between the two groups was based on parametric Student’s two-sample *t*-test, or Cochran–Cox test or nonparametric Mann–Whitney test as appropriate. Student’s paired *t*- or Wilcoxon’s test was used to compare continuous variable differences between the baseline and the end of observation period. We used the Shapiro–Wilk test to assess normal distribution of the variables.

The differences in proportions between categorical data were examined with the use of the Chi-square test with Yates’s correction or Fisher’s exact test as appropriate.

The Kaplan–Meier curves were performed to visualize cumulative probability of events. We used log-rank test to compare cumulative events rates. The assessment of any potential influence of covariates was performed using a Cox proportional hazards regression models.

Statistical analysis was performed using the SAS 9.2 statistical package (SAS Institute Inc., Cary, NC, USA).

## Results

**Fig. 1:**
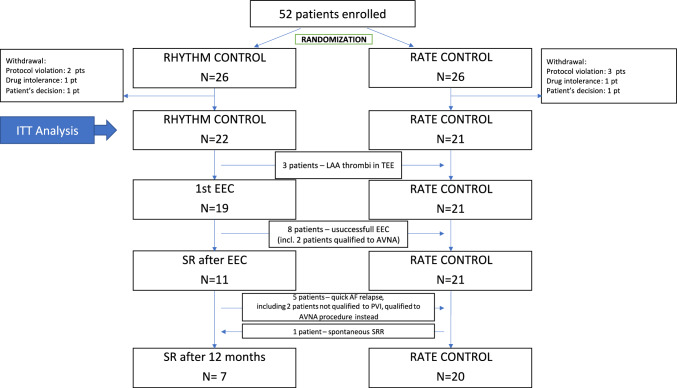
Study flow chart including reasons for withdrawal from the study and cross-overs. *AF* atrial fibrillation, *AVNA* atrioventricular node ablation, *EC* external electric cardioversion, *ITT* intention to treat, *LAA* left atrial appendage, *PP* per protocol, *pt* patient, *pts* patients, *PVI* pulmonary vein isolation, *SR* sinus rhythm, *SRR* sinus rhythm resumption, TEE – transoesophageal echocardiography

Among 52 patients enrolled during the study enrollment period (November 2013–January 2018), the final analysis was performed on 43 patients (97.7% males). Figure 1 presents the study flow chart with the reasons of drop-outs and cross-overs. The mean age of the study participants was 68.4 years. Mean baseline BiVp% was 82.4% and the current AF episode lasted more than 2 years. The echo examination on the enrollment visit revealed mean baseline left ventricular ejection fraction (LVEF) of 30 ± 8%, left atrium area (LAA) 33 ± 7 cm^2^ and the maximal oxygen uptake (VO2max) of 14 ± 5 mL/(kg*min). The detailed baseline characteristics of the study population is presented in Table 1.

**Table 1:** Baseline characteristics of the studied groups

	Rhythm control *N* = 22	Rate control *N* = 21	*P*
*Basic characteristics*			
Age	68.6 ± 8.7	68.2 ± 8.1	0.881
Sex (% males)	21 (95.4%)	21 (100%)	0.333
Time of current AF paroxysm (months)	21.3 ± 14.1	29.7 ± 23.0	0.171
NYHA III	9 (42.9%)	7 (33.3%)	0.525
HF etiology (ICM/DCM/other)	11/11	10/11/1	
*Concomitant diseases*			
DM	12 (54.5%)	5 (23.8%)	0.039
Hypertension	14 (63.6%)	11 (52.4%)	0.455
Stroke	1 (4.5%)	2 (9.5%)	0.607
COPD	3 (13.6%)	2 (10%)	0.716
CKD	5 (22.7%)	1 (4.8%)	0.185
Coronary disease	11 (50%)	14 (70%)	0.187
MI	10 (47.6%)	7 (33.3%)	0.346
*Pharmacotherapy*			
ACE-I	15 (68.2%)	18 (85.7%)	0.281
ARB	3 (13.6%)	2 (9.5%)	1.00
BB	21 (95.4%)	20 (95.2%)	1.00
MRA	15 (68.2%)	19 (90.5%)	0.132
Diuretics	19 (86.4%)	21 (100%)	0.223
Amiodarone (before enrollment)	6 (27.3%)	5 (23.8%)	0.795
Digoxin	13 (59.1%)	10 (47.6%)	0.451
*Baseline ECHO*			
LA (mm)	52.8 ± 6.2	51.1 ± 7.4	0.422
LAA (cm^2^)	32.5 ± 6.6	34.5 ± 8.0	0.399
LVEDD (mm)	64.1 ± 9.3	65.5 ± 5.8	0.562
LVEF (%)	30.4 ± 9.2	30.1 ± 7.7	0.909
*Baseline CPX*			
VO2max (ml/min/kg)	14.7 ± 5.7	12.8 ± 4.0	0.260
*Baseline QoL*			
MLHFQ (points)	40.6 ± 21.8	34.2 ± 21.7	0.347
*Baseline CRT examination*			
BiVp% (%) [95% CI]	84.0 [78.0–91.2]	83.7 [79.0–90.0]	0.952

*ACE-I* angiotensin-converting enzyme inhibitors, *ARB* angiotensin receptor blockers, *AF* atrial fibrillation, *BiVp% *percentage of biventricular paced beats, *CKD* chronic kidney disease, *COPD* chronic obstructive pulmonary disease, *CPX* cardiopulmonary exercise test, *CRT* cardiac resynchronization, *DCM* dilated cardiomyopathy, *DM* diabetes mellitus, *ICM* ischemic cardiomyopathy, *LA* left atrium dimension, *LAA* left atrium area, *LVEF* left ventricular ejection fraction, *LVEDD* left ventricular end-diastolic dimension, *MI* history of myocardial infarction, *MLHFQ* Minnesota Living with Heart Failure Questionnaire, *MRA* mineralocorticoids receptors blockers, *QoL* quality of life, *VO2max* maximal oxygen uptake

There were 22 patients assigned to the rhythm control arm (RHYTHM). The EC was performed in 19 of them (Fig. 1). The EC resulted in sinus rhythm restoration in 11 of 19 patients (58%) regardless of the amiodarone pre-treatment. After 12 months, SR was maintained only in 27% and 32% patients from the rhythm control group (*n* = 6), according to the ITT and PP principles, respectively. None of the rhythm control group patients had AF ablation performed.

The rate control group (RATE) comprised 21 patients. During the study follow-up period, AVNA procedure was performed in 8 patients (18.6%): 4 of them were originally assigned to the rate control (19%) and 4 were assigned to the rhythm control arm at first (18%) but they migrated to the rate control strategy after cardioversion failure or quick AF relapse. One patient from the RATE group experienced spontaneous sinus rhythm resumption which took place during the 10th month of the follow-up.

The treatment arms did not differ significantly when it comes to baseline characteristics, HF etiology, baseline ECHO, CPX and QoL assessment, except for the diagnosis of diabetes mellitus, which was more frequent in the RATE group (Table 1). The mean baseline BiVp% in the RHYTHM and the RATE groups was 84% and 83.7%, respectively (*p* = 0.856).

**Fig. 2:**
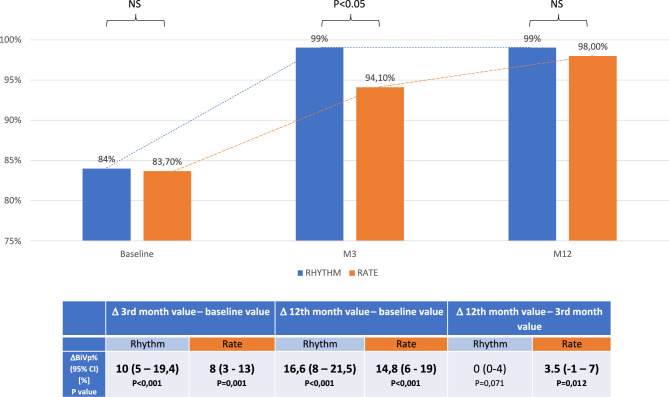
BiVp% changes during the follow-up, intention-to-treat analysis. The values in the table are presented as a median with the 95% confidence interval. In both groups, BiVp raised significantly and reached desired level exceeding 95% at the end of follow-up. However, in the RHYTHM group, BiVp raise was immediate (restricted to the first 3 months of follow-up), whereas in the RATE group, it raised continuously throughout the whole follow-up period. BiVp%—biventricular paced beats percentage, *CI* confidence interval, *M0* baseline visit, *M3* visit after 3 months of follow-up, *M12* visit after 12 months of follow-up, *NS* not significant, *P* probability, *Rate* rate control group, *Rhythm* rhythm control group

During the follow-up period, a significant BiVp% increase was observed in both treatment arms reaching 99% in the RHYTHM and 98% in the RATE groups at the 12-month visit. The 12-month BiVp% difference between the treatment arms was not significant. However, BiVp% raised more quickly in the RHYTHM group reaching 99% in comparison to 94.1% in the RATE group at 3 months (*p* < 0.05). The BiVp% changes during the follow-up are presented in Fig. 2.

**Fig. 3:**
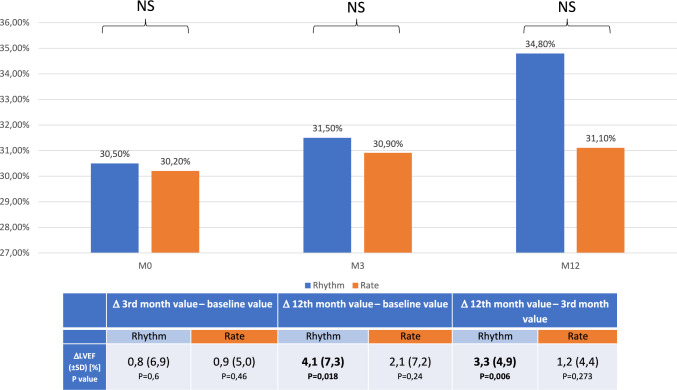
LVEF changes during the follow-up, intention-to-treat analysis. There were no significant differences in LVEF at baseline, after 3 and 12 months (graph). However, in the Rhythm control group, significant rise of the LVEF was observed. LVEF rise was not immediate after SR restoration but delayed (no significant change during first 3 months of observation) (table). *LVEF* left ventricle ejection fraction, *M0* baseline visit, *M3* visit after 3 months of follow-up, *M12* visit after 12 months of follow-up, *NS* not significant, *P* probability, *Rate* rate control group, *Rhythm* rhythm control group, *SD* standard deviation

**Fig. 4:**
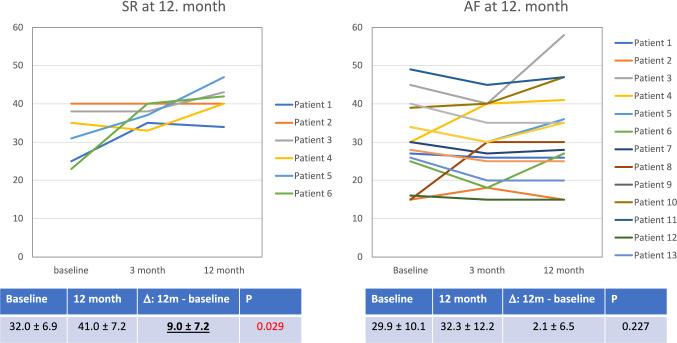
Plots and tables showing LVEF change in each patient from the RHYTHM group, according to SR rhythm maintenance on the 12-month visit. Patients who maintained sinus rhythm after cardioversion experienced LVEF rise, whereas in patients with AF relapse, LVEF rise was not observed. Values in the tables are expressed as means ± standard deviation (SD). *AF* atrial fibrillation, *LVEF* left ventricular ejection fraction, *SR* sinus rhythm, *P* probability

There were no significant differences in the mean values of the secondary end-points after 12 months between the groups (Table 2). However, after the correction for the baseline values, a significant increase of LVEF was observed in the RHYTHM group (Fig. 3 and Table 3). The LVEF rise was driven and restricted to these patients from the RHYTHM group who maintained sinus rhythm through the whole follow-up (Fig. 4). We also observed an increase of the quality of life in the RHYTHM group, although the QoL change was statistically significant only in the first 3 months of the follow-up.

**Table 2:** Intention-to-treat analysis of the prespecified end-points

	Rhythm *N* = 22	Rate *N* = 21	*P*
LA (mm)	50.7 ± 7.2	52.6 ± 8.7	0.442
LAA (cm^2^)	32.5 ± 7.8	38.9 ± 10.7	0.060
LVEDD (mm)	63.3 ± 7.2	63.8 ± 7.1	0.825
LVEF (%)	34.8 ± 11.2	31.0 ± 9.9	0.271
VO2max (ml/min/kg)	15.7 ± 4.8	13.5 ± 5.1	0.178
MLHFQ (points)	31.4 ± 22.5	36.7 ± 22.4	0.489
BiVp% (%) [95% CI]	99.0 [97.3–99.8]	98.0 [94.0–99.0]	0.144

*BiVp*% percentage of biventricular paced beats, *LA* left atrium dimension, *LAA* left atrium area, *LVEF* left ventricular ejection fraction, *LVEDD* left ventricular end-diastolic dimension, *LVESD* left ventricular end-systolic dimension, *MLHFQ* Minnesota Living with Heart Failure Questionnaire, *VO2max* maximal oxygen uptake

The values are expressed as means with ± standard deviation or as medians with 95% confidence interval—as appropriate

**Table 3:** Intention-to-treat analysis of the changes of analyzed end-points in time

	Rate *N* = 21	Rhythm *N* = 22	Difference [95% CI]^†^	*p*^‡^
*Δ 3-month value – baseline value*				
LAA (cm^2^)	2.32 (– 0.52; 5.17)	2.31 (– 0.73; 5.35)	0.01 (– 4.18; 4.21)	0.994
LVEDD (mm)	0.86 (– 1.14; 2.86)	– 0.06 (– 2.06; 1.94)	0.92 (– 1.92; 3.76)	0.515
LVEF (%)	1.27 (– 1.30; 3.83)	0.89 (– 1.61; 3.39)	0.38 (– 3.20; 3.96)	0.831
VO2max (ml/min/kg)	– 0.06 (-2.59; 2.48)	– 0.07 (– 2.79; 2.65)	0.01 (– 3.78; 3.80)	0.994
MLHFQ (points)	3.9 (– 5.5; 13.4)	**− 8.8 (– 17.1; – 0.5)**	12.7 (– 0.1; 25.5)	0.052
*Δ 12-month value – baseline value*				
LAA (cm^2^)	2.08 (– 1.07; 5.24)	0.14 (– 2.80; 3.09)	1.94 (– 2.41; 6.29)	0.369
LVEDD (mm)	– 1.93 (– 4.62; 0.78)	– 0.82 (– 3.38; 1.74)	– 1.11 (– 4.84; 2.62)	0.551
LVEF (%)	2.01 (– 1.47; 5.50)	**4.13 (0.90; 7.36)**	– 2.12 (– 6.87; 2.64)	0.372
VO2max (ml/min/kg)	0.92 (– 0.70; 2.54)	1.15 (– 0.47; 2.77)	– 0.23 (– 2.54; 2.09)	0.842
MLHFQ (points)	0.88 (– 6.93; 8.68)	– 5.81 (– 13.8; 2.22)	6.69 (– 4.51; 17.89)	0.233

*LAA* left atrium area, *LVEF* left ventricular ejection fraction, *LVEDD* left ventricular end-diastolic dimension, *MLHFQ* Minnesota Living with Heart Failure Questionnaire, *VO2max* maximal oxygen uptake

^†^The difference between the Rate and the Rhythm groups

^‡^*P* value for the RATE vs RHYTHM difference

The values are presented as mean change with the 95% confidence interval (CI), corrected for the baseline values. The differences in time that are statistically significant are marked in bold

The groups did not differ in the occurrence of adverse events. Amiodarone-related adverse events occurred in 8 patients including one patient with high INR levels that eventually led to gastrointestinal hemorrhage, one patient with hypothyroidism and one with the suspicion of hyperthyroidism. In the remaining 5 cases, amiodarone was discontinued due to its intolerance (without any serious adverse events). The incidence of ventricular arrhythmias and CRT-D interventions was low and also similar in the treatment arms.

There was strong correlation between the LVEF raise in the RHYTHM control group and sinus rhythm maintenance (Fig. 4).

## Discussion

This study is the first randomized trial that compares rhythm and rate control strategies in treatment of persistent atrial fibrillation dedicated to patients with CRT—a specific population in which AF treatment may have a direct influence on their heart failure treatment (mediated by the CRTs BiVp% increase). To the best of authors’ knowledge, it is the first attempt to fill in, at least partially, the gaps in evidence on the efficacy of CRT in persistent AF patients, which are still listed in the latest ESC guidelines [6, 13].

Regardless of the fact that both of the treatment strategies resulted in similar and marked increase of BiVp% exceeding 95%, an increase of LVEF was observed only in the rhythm control group and it was restricted to patients who maintained sinus rhythm through the whole follow-up.

There may be at least two reasons for the lack of effectiveness of the rate control strategy observed in our study.

The first one is that although high, the BiVp% in this group was not high enough. Such hypothesis seems to be reasonable in view of the APAF-CRT study results in which CRT implantation and AVNA in patients with heart failure and persistent atrial fibrillation lasting more than 6 months has led to mortality reduction and lower risk for HF hospitalizations and had to be stopped prematurely for efficacy [15]. However, it should be underlined that the APAF-CRT study population differed significantly from the one observed in our study. Contrary to Pilot-CRAfT patients, the APAF-CRT population comprised of patients that did not met standard CRT criteria (narrow QRS + and at least one hospitalization for heart failure irrespective of the ejection fraction). According to large observational studies of Hayes [10] and Ousdigian [11], the BiVp% cutoff value which guarantees the best effectiveness of CRT (resulting in the biggest mortality reduction) is > 98%. Indeed, 71% of patients from the RHYTHM group (and 87.5% of those who had SR at 12 months) had BiVp ≥ 98% in comparison to only 56% of patients with BiVp ≥ 98% in the RATE group. However, the observational works of Tolosana, dedicated to patients with BiVp reduction associated with permanent atrial fibrillation suggest that no additional effect in further BiVp% increase above 90–95% is observed, irrespective of the fact whether patients were in SR or had the ventricular rate controlled by drugs or by AVNA [16, 17]. As a result, the current ESC Guidelines on Cardiac Pacing suggest performing AVNA only in patients with BiVp lower than > 90–95% [6]. It has been shown that CRT effectiveness starts to rise beginning with the values of BiVp% exceeding 90% [10, 11], so the evident increase of BiVp% in the RATE control group in our study should have had a positive influence on the factors assessed.

Another possible explanation for a better outcome of the rhythm control may be a positive effect of sinus rhythm maintenance itself, irrespective of the BiVp%. This hypothesis is supported by the fact that LVEF rise was restricted predominantly to patients who maintained sinus rhythm during whole follow-up period. A significant rise in LVEF after sinus rhythm restoration suggest the presence of arrhythmia/atrial fibrillation-mediated cardiomyopathy (AMC) in this group of patients. AMC concerning further deterioration of LV function and exacerbation of HF symptoms related to the occurrence of arrhythmia/atrial fibrillation in patients with already impaired LV function due to underlying structural heart disease remains still an underestimated, potentially reversible cause of HF exacerbation. Indeed, successful elimination of arrhythmia in AMC patients leads increase of LVEF as it was shown in the CAMERA-MRI study and the improvement of in the quality of life [18, 19]. Sinus rhythm control may positively affect the prognosis of patients with HF as it was proven in the CASTLE-AF, the AMICA and, recently, in the CASTLE HTx trials [20–23]. What is important, the rhythm control strategy implemented in the aforementioned trials was characterized by significant percentage of AF relapses proving limited efficacy in maintaining sinus rhythm in the HF patients’ population as it was shown in our study. In the AMICA trial, a sinus rhythm was observed in 73.5% of patients in the ablation group and 50% in the best medical therapy group after 12 months of the follow-up. Even greater number of AF relapses was observed in rhythm control arm of the CASTLE HTx trial—the lack of AF was observed only in 5% of the ablation group after 1 year of follow-up, even though the AF burden was significantly reduced in the ablation group suggesting that it is more AF burden reduction than a complete AF elimination that has prognostic effect in the HF patients.

Contrary to our study, CRT patients constituted the minority in the CASTLE-AF, AMICA and CATLE-HTx trials. However, the similarity of our study results with these of AMICA suggests that the beneficial effect of sinus rhythm maintenance in HF patients is comparable between CRT and non-CRT patients.

Although beneficial, rhythm control was achieved in a very limited number of the study participants. The main reason for that may be a very long duration of AF in our group. Other factors limiting the efficacy of antiarrhythmic treatment in our study were the participants’ age, serious LA enlargement and serious LVEF impairment that were all generally more advanced in comparison to both the CASTLE-AF and the AMICA trials [21, 22].

Recently, new forms of cardiac resynchronization that is: left bundle branch area pacing (LBBAP) and left bundle branch-optimized cardiac resynchronization (LOT-CRT) have emerged becoming a potential alternative to the standard biventricular CRT [24, 25]. However, their ability in overcoming interventricular conduction abnormalities is still inherently connected with the amount of effectively captured paced beats, making the results of our study potentially applicable in LBBAP and LOT-CRT-treated patients with AF as well.

### Limitations

The main limitation of the study is a low number of participants. Though, interpretation of the study secondary end-points’ results should be regarded as hypothesis generating. In order to improve the enrollment rate, the Steering Committee of the trial decided to prolong the enrollment phase, invited another high-volume center and changed the minimal duration of current AF paroxysms from at least 1 year to 6 months. Regardless of these measures, the study did not reach the prespecified number of 27 participants in each study arm [14].

The study population consisted of vast majority of males (97%) which makes interpolation of our results to women population impossible.

Another limitation is a heterogeneity within the strategies applied, while only small number of patients from the RATE group underwent AVNA and PVI CA was rarely performed in the RHYTHM group. The reason for rare AVNA use was that in majority of RATE patients, BiVpace > 95% was achieved with drugs only. As it comes to pulmonary vein isolation, it was not offered to many patients due to their unfavorable characteristics hindering ablation efficacy (discussed above). It has to be underlined that the vast majority of patients were followed up before the publication of CASTLE-AF and the subsequent trials, which led to a broader use of CA in severe HF patients. Nevertheless, a more strict protocol may have diminished heterogeneity in the groups.

We examined BiVp% values assessed by CRT devices, which may be artificially higher than the real effectively captured BiVp beats percentage due to the inability to rule out fusion and pseudo-fusion beats [7]. However, we routinely switched-off algorithms that trigger LV pacing after RV sensed beats, which was not the case in the studies of Tolosana and could lead to overestimation of BiVp% in their no-AVN ablation group [16, 17].

## Conclusions

Our study remains the first and single attempt to assess the effectiveness of rate and rhythm control strategies in patients with long-standing AF treated with CRT in a prospective and randomized manner. The study results suggest that both the rate and the rhythm control strategies may lead to a comparable increase of BiVp% in these patients. Intensification of pharmacological treatment to control ventricular rate in many cases may be enough to reach 95% of BiVp goal. Irrespective of the lack of statistic differences in BiVp% between the studied arms, only the RHYTHM control strategy led to an increase of left ventricular ejection fraction, suggesting that limited CRT efficacy in AF is associated more with the presence of AF itself rather than simple BiVp% reduction. However, conservative RHYTHM control strategy remains very limited in efficacy in these patients. Nevertheless, in the few who maintained sinus rhythm, a marked LVEF improvement was observed to such an extent that it led to a statistical increase of LVEF in the whole RHYTHM control arm. Further trials are needed to confirm or disprove our findings. In view of our study results, the search prognostic factors positively affecting sinus rhythm maintenance in this subpopulation of patients is of prime importance.

## Data Availability

The data that support the findings of this study are available from the corresponding author, [JBC], upon reasonable request.
